# S04-1: The PA-EPI: Physical Activity Environment Policy Index

**DOI:** 10.1093/eurpub/ckae114.211

**Published:** 2024-09-26

**Authors:** Catherine Woods

**Affiliations:** Physical Activity for Health Research Centre (PAf), Health Research Institute, Department of Physical Education and Sport Sciences, University of Limerick

## Abstract

**Purpose:**

The ‘Physical Activity Environment Policy Index’ (PA-EPI) is a monitoring tool and framework to assess the extent of implementation of government policies and actions for creating a healthy physical activity (PA) environment.

**Methods:**

The PA-EPI is conceptualised as a two-component ‘policy’ and ‘infrastructure support’ framework. The two components comprise eight policy and seven infrastructure support domains. The policy domains are education, transport, urban design, healthcare, public education (including mass media), sport-for-all, workplaces and community. The infrastructure support domains are leadership, governance, monitoring and intelligence, funding and resources, platforms for interaction, workforce development, and health-in-all-policies. Forty-five ‘good practice statements’ (GPS) or indicators of ideal good practice within each domain concludes the PA-EPI. There is an eight-step process for conducting the PA-EPI which involves developing an evidence document, working with government officials to validate the evidence and benchmarking this evidence with country special PA-EPI councils representing stakeholders and citizens.

**Results:**

Two countries –Ireland and the Netherlands – have completed the PA-EPI. An additional five countries–Germany, Lithuania, Poland, Romania and Slovenia - are in the process. The PA-EPI scores for Ireland and the Netherlands range from little or no evidence of implementation (0) to full implementation (4) across each of the eight policy and seven infrastructure domains. In Ireland of the 45 PA-EPI indicators, one received an implementation rating of ‘very little’, 25 a rating of ‘low’, 19 ‘ medium’ and no indicator was scored ‘full implementation’. For example, indicator SP01 ‘sport policies prioritise investment in initatives that target the least active’ received a medium, while ‘regulations in healthcare include routine screening for physical activity’ was rated to have a low implementation status. Recommendations for addressing critical implementation gaps are being addressed by each country.

**Conclusions:**

The PA-EPI can help policymakers determine where their country is now in relation to the implementation of PA policies and what is possible to change. It also provides pathways for policymakers on how to reach goals to address critical implementation gaps and a mechanism for documenting progress. In time, benchmarks will be established by governments at the forefront of creating and implementing policies to address population levels of inactivity.

**Funding source:**

Policy Evaluation Network (PEN) a Joint Programming Initiative (JPI) “A Healthy Diet for a Healthy Life” and ERA4Health; GA N° 101095426 of the EU Horizon Europe Research and Innovation Programme

